# Seasonal variations in carbon, nitrogen, and phosphorus of *Pinus yunnanenis* at different stand ages

**DOI:** 10.3389/fpls.2023.1107961

**Published:** 2023-05-09

**Authors:** Siyi Liang, Tian Tan, Danzi Wu, Chaojun Li, Huiqing Jing, Junwen Wu

**Affiliations:** College of Forestry, Southwest Forestry University, Kunming, Yunnan, China

**Keywords:** ecological chemometry, stand age, organs, different seasons, Pinus yunnanensis, allometry

## Abstract

The seasonal variations in carbon (C), nitrogen (N), and phosphorus (P) at the organ level of *Pinus yunnanenis* during different season are poorly understood. In this study, the C, N, P, and their stoichiometric ratios in various organs of *P. yunnanensis* during the four seasons are discussed. The middle and young aged *P. yunnanensis* forests in central Yunnan province, China were chosen, and the contents of C, N, and P in fine roots (<2 mm), stems, needles, and branches were analyzed. The results showed that the C, N, P contents and their ratios in *P. yunnanensis* were significantly influenced by season and organ, less affected by age. The C content of the middle-aged and young forests decreased continuously from spring to winter, whereas N and P first decreased and then increased. No significant allometric growth relationships were observed between P-C of the branches or stems in the young and middle-aged forests, whereas a significant allometric growth relationship existed for N-P of needles in the young stands, indicating that the P-C and N-P nutrient distribution patterns shows different trends in the organ level in different age stands. The pattern of P allocation between organs shows differences in stand age, with more allocation to needles in middle-aged stands and more allocation to fine roots in young stands. The N:P ratio in needles was less than 14, indicating that *P. yunnanensis* was mainly limited by N and increasing the application of N fertilizer would be beneficial for the productivity of this stand. The results will be helpful to nutrient management in *P. yunnanensis* plantation.

## 1 Introduction

Plant growth and development is a process of accumulation and mutual coordination of the ratio of elements, so elements are important indicators of plant growth, particularly carbon (C), nitrogen (N), and phosphorus (P) (Sardans et al., 2012). C is the basic structural substance that constitutes the plant body and provides energy for plant physiological activities (Sardans and Peñuelas, 2012). N is a key element that plays an important role in the biosynthesis of amino acids, proteins, nucleic acids, and other substances, and in enhancing photosynthesis. P is an essential component of nucleic acids and enzymes (Dawson and Curran, 1998). The C, N, and P composition of plants at each season determine ecosystem processes, and their ratios play an important role in indicating energy flow, material cycling, and nutrient limitations in the ecosystem. Because of the different plant C assimilation and nutrient uptake pathways, it is generally believed that C does not limit plant growth under natural conditions, so changes in N and P are the main factors affecting the C:N and C:P ratios (Hedin, 2004). For example, the C:N and C:P ratios represent the ability of plants to assimilate C during nutrient uptake and the efficiency of nutrient use by plants (Herbert et al., 2003); the N:P ratio is a key indicator of community structure and function and can be used to indicate nutrients that limit productivity (Tessier and Raynal, 2003). The soil C:N:P ratio reflects the nutrients contained in the soil and its elemental reserves, leaf content affect the nutrient status of plants (Li et al., 2012; Batjes, 2014). Therefore, it is important to explore C, N, and P contents in plants and their ratios.

The nutrient allocation between different organs represents the influence of external factors on plant growth (Ma et al., 2016). Allometric growth is a phenomenon with different relative growth rates of two traits in an organism (Weiner, 2004).The uptake and allocation of elements can be reflected by allometric growth relationships, which can characterize the stoichiometry-limiting strategies of plants in different habitats (Niklas et al., 2005). Enquist et al. (2007) noted that the allometric exponent between sapling metabolic rate and individual size was 1, which became 3/4 as the plant growth. Cheng et al. (2010) also stated that the allometric exponent was 1 for small saplings and 0.82 for larger plant individuals. At present, research on forest ecosystems has focused on C, N, P and their ecological stoichiometry in forest communities, populations, and single vegetation organs. However, the interactions and differences in the distribution of nutrient elements between individual vegetation organs are still unclear, and whether the mechanisms of adaptation of each organ to the environment have any influence on the nutrient cycling of vegetation remains unconfirmed.

The C, N, and P ratios in plants are influenced by environmental conditions (e.g. temperature, altitude, and moisture), plant age, and the growing season (Han et al., 2005; Sardans et al., 2011). When stoichiometry is used as a functional feature to describe community structure, stoichiometric differences within individual plants or across ages cannot be ignored (McGill et al., 2006; Cianciaruso et al., 2009; Ågren and Weih, 2012). Zhang et al. (2013) showed that C:N and C:P ratios of grassland species in Inner Mongolia were influenced by sampling date and leaf age. Neves et al. (2022) studied legume and non-legume trees showing that litter dynamics and nutrient inputs in secondary seasonal dry tropical forests are influenced by season and the dominance of different functional groups. Ugawa et al. (2018) showed that the N content of fine roots also decreases with the development of a subalpine coniferous forest stand. Cheng et al. (2018) analyzed biomass and nutrient data measured continuously over 40 years in *Cunninghamia lanceolata* stands and concluded that organ nutrient concentrations gradually decrease as the stand aged. Zhang et al. (2018) studied nutrient uptake in *Metasequoia glyptostroboides* forests of coastal areas in China. They showed that the C:P and N:P ratios were significantly lower in young stands than in middle-aged stands. Changes in nutrient (C, N, and P) concentrations reflect nutrient uptake and utilization of different aged stands and their environmental adaptability (Wright and Westoby, 2003; Yang and Luo, 2011). Plants have a self-regulatory adaptation strategy to environmental change, especially in their physiology changes with the season, which, in turn, affects the C, N, and P stoichiometry (Rivas-Ubach et al., 2012). Seasonal changes have significant effects on leaf C, N, and P stoichiometry in *Quercus suber L*. (Orgeas et al., 2003) and Dunhuang, *Phragmites australis* (Liu et al., 2020). The N:P ratio of plant leaves as a criterion for environmental limiting elements is not only a key indicator for determining community structure and function, but also a nutrient element indicator that plays a limiting role in productivity (Elser et al., 2000). The relationships between the changes in nutrient element (C, N, and P) concentrations and the ratios in organs at different season and seasons are key to plantation management and fertilization practices, so it is important to explore the relationships between age and seasonal changes and plant adaptability to various environments.

*Pinus yunnanensis* is a major pioneer tree species in the forests of southwest China and an important afforestation species with great economic and ecological values. This species is distributed from 710 to 3,320 m above sea level and is concentrated in the elevation range of 1,500 to 2,500 m (Wang et al., 2013). Most studies on *P. yunnanensis* forests have focused on population structural characteristics (Tang et al., 2020), seed adaptability (Su et al., 2019), ecosystem functions (Huang et al., 2019), and their response to climate change (Yang et al., 2022). However, the pattern of the stoichiometric characteristics of *P. yunnanensis* with season and age remains unclear. In this study, we compared the seasonal variations in C, N, and P stoichiometry of each organ in *P. yunnanensis* plantations in central Yunnan Province, and addressed the following: (1) The age and seasonal variation of the ecological stoichiometry in each organ of *P. yunnanensis* in middle and young-age plantations. (2) The allometric growth relationships among the organs of middle and young stands of *P. yunnanensis*. The results of this study will enrich the chemometrics of *P. yunnanensis* and provide a theoretical basis for *P. yunnanensis* plantation management.

## 2 Materials and methods

### 2.1 Site description

The experimental site was located in the state-owned Garden Forest in Yiliang County(26°11′-26°25′N, 101°27′-101°28′E), Kunming City, Yunnan Province, at an altitude of 1,300–2,800 m. The entire experimental site is influenced by a subtropical monsoon climate, the annual precipitation is clearly divided into dry and wet seasons, the rainy season from May to October, accounts for about 85% of the annual precipitation, while the dry season, from November to April, accounts for only about 15% of the annual precipitation. The number of hours of sunlight is 2,177.3, the frost-free period is 260 days.

Middle and young monoculture stands of *P. yunnanensis* with a uniform vegetation distribution and strong regional representation were selected as sample plots in the study area (24˚54′4″N, 103˚5′9″E). A total of 6 standard plots (30 × 30 m) were randomly set up, such as 3 in the middle-aged forest and 3 in the young forest, The distance between each sample plot is more than 30m. The plots were distributed on the upper and middle slopes, with an average elevation of 2,343 m. Three trees were selected as standard trees in each plot, with an average diameter at breast height of 20.44 cm and an average height of 12.59 m in the middle-aged forest, and an average diameter at breast height of 12.44 cm and an average height of 7.25 m in the young forest. The stand density is 1058 tree**·** hm^-2^ for young stands of 15 years and 753 tree**·** hm^-2^ for middle-aged stands of 25 years. The soil type was red loam with 27.1 g· kg^-1^ organic carbon, 1.27 g· kg^-1^ total nitrogen and 0.57 g· kg^-1^ total phosphorus.

### 2.2 Sample collection

We sampled in May 29th (spring), August 1st (summer), November 9th (autumn) and January 20th (winter) of 2021 and 2022, respectively. The needles in the mid-level of the canopy were selected for this study to represent the needles in the entire canopy layer. Three twigs of first-order branches (branches directly connected to the trunk were defined as first-order branches, second-order branches were directly connected to first-order branches, and those directly connected to second-order branches were third-order branches) were taken from each sample tree to eliminate sampling errors in needle nutrient content due to differences in the orientation of the branches. Three tertiary branches were randomly cut on selected primary branches of each sample tree using high branch shears, and the tertiary branches cut in 4 directions were evenly mixed, and the needles of each age class were picked separately according to the branch, with 1 mixed needle sample for each of the three sample trees in each plot. Stems were collected by the increment borer method, and trunk samples were collected with increment borer at a position about 1.5 m above the ground on selected standard wood (Takahashi, 2008). The harvested fine root (<2 mm), stem, and needle samples were bagged and used to determine the C, N, and P contents of the plant needles and roots.

The plant samples were transported to the laboratory, rinsed, and placed in an oven at 105°C to prevent enzymatic carbohydrate reactions, and then dried at 80°C to a constant dry weight, crushed with a pulverizer, and sealed for storage. Plant whole C content was determined using the potassium dichromate plus dilution heating method, Whole N content was determined by the naive colorimetric method (Bao, 2000), and whole P was determined by the molybdenum antimony anti-colorimetric method (Sparks et al., 2020). The results are expressed as nutrient content per unit mass (g·kg^-1^).

### 2.3 Data analysis

Data pre-processing, plotting, and statistical analysis were performed in Microsoft Excel 2013 (Microsoft Inc., Redmond WA, USA), GraphPad 8.0, (GraphPad Software Inc., La Jolla, CA, USA), and SPSS 22.0 (SPSS Inc., Chicago, IL, USA) software, respectively. The data were tested for normality and homogeneity of variance prior to analysis of variance. Descriptive statistics were used to analyze the characteristics of the C, N, and P contents and their stoichiometric ratios in the organs of *P. yunnanensis*, and least-significantly different multiple comparison tests and one-way analysis of variance (ANOVA) were used to detect the differences in C, N, and P contents and their stoichiometric ratios among the different organs and growing seasons. Multi-factor ANOVA in the GLM model was used to analyze the effects of different seasons and organs on the C, N, and P contents and their stoichiometric ratios. Pearson’s correlation analysis was performed to determine the correlations between the stoichiometric parameters in different organs. The coefficient of variation (CV) was calculated using the formula CV = standard deviation/mean × 100%. Allometric growth reflects the variation in two attributes of an organism as shown by growth and development, and the common formula is: y = ax^b^, where a and b are constants, and x and y are attributes of the organism, such as the C, N, and P contents. The allometric growth relationship function is usually expressed in a logarithmic form as log(y) = b·log(x) + log(a), where b is the slope of the line, and the allometric exponent (Niklas, 1994). b = 1, indicates that the two elements are in equal proportion in plant organ, and b > 1 or b < 1 means that the two elements are in allometric proportional relationship. Standardized principal axis regression analysis (SMA) was used to calculate the slope, b, and compare the differences between the slope and 1. The SMA analysis was performed using the SMATR module in R language (Warton et al., 2012).

## 3 Results

### 3.1 Analysis of the overall variations in C, N, and P contents and their stoichiometric ratios in *P. yunnanenis*

Different seasons and organs had highly significant effects on the C, N, and P contents, and the interaction between age and growing season only had highly significant effects on N content, both season and organ had significant effect on N and P contents, respectively. The season had a significant effect on the N: P ratio and different organs had significant effects on the C: P ratio, while the interaction between different growing seasons and organs was highly significant for all stoichiometric indicators except for the C:P or N:P ratio. (**Table 1**). Season, age and organ, and the interaction of the three had a highly significant effect on all indicators except N:P. The interaction of age, season and organ had a highly significant effect on all indicators except N:P, only season and organ had a significant effect on N:P.

**Table 1:** Multi-factor ANOVA on the effects of age, season, and organ on C, N, and P concentrations and their ratios in *P. yunnanensis*.

Variable	Dependent variable	Sum of squares	df	Mean square	F-value
Season(S)	C	720569.331	3	240189.777	56.362**
	N	188.65	3	62.883	182.535**
	P	50.842	3	16.947	137.102**
	C:N	319263867	3	106421289	1582.346**
	C:P	785721405.5	3	261907135.2	63.881**
	N:P	258.078	3	86.026	4.373*
Age(A)	C	762.451	1	762.451	0.179
	N	0.221	1	0.221	0.642
	P	0.181	1	0.181	1.464
	C:N	100153.858	1	100153.858	1.489
	C:P	1348463.792	1	1348463.792	0.329
	N:P	17.99	1	17.99	0.914
Organ(O)	C	94759.373	3	31586.458	7.412**
	N	147.998	3	49.333	143.201**
	P	16.826	3	5.609	45.373**
	C:N	81925685.02	3	27308561.67	406.043**
	C:P	202204110	3	67401370.01	16.44**
	N:P	175.083	3	58.361	2.967*
Age×Season (A×S)	C	3536.981	3	1178.994	0.277
	N	9.712	3	3.237	9.397**
	P	0.272	3	0.091	0.734
	C:N	251277.363	3	83759.121	1.245
	C:P	5177179.987	3	1725726.662	0.421
	N:P	58.066	3	19.355	0.984
Age×Organ (A×O)	C	19445.532	3	6481.844	1.521
	N	3.504	3	1.168	3.391*
	P	0.455	3	0.152	1.228
	C:N	169868.186	3	56622.729	0.842
	C:P	6450825.028	3	2150275.009	0.524
	N:P	62.846	3	20.949	1.065
Season×Organ (S×O)	C	254438.308	9	28270.923	6.634**
	N	50.687	9	5.632	16.348**
	P	9.762	9	1.085	8.775**
	C:N	122034774	9	13559419.33	201.611**
	C:P	296511548	9	32945727.56	8.036**
	N:P	138.516	9	15.391	0.782
Age×Season×Organ (A×S×O)	C	320690.195	24	13362.09	3.15**
	N	67.971	24	2.832	8.312**
	P	11.915	24	0.496	4.056**
	C:N	122581186.7	24	5107549.446	73.883**
	C:P	351416806	24	14642366.92	3.593**
	N:P	396.545	24	16.523	0.833

*P < 0.05, **P < 0.01

### 3.2 Characteristics of the C, N, and P contents and their stoichiometric ratios in various organs of *P. yunnanensis* at young and middle-aged stands

The mean values of C, N, and P contents and the stoichiometric ratios of fine roots in the young stands were higher than those in the middle stands (**Table 2**). The CV is an absolute value reflecting the degree of dispersion of the data; CV ≤ 10% represents weak variation, 10% < CV < 100% represents moderate variation, and CV ≥ 100% represents strong variation.

**Table 2:** Statistical parameters of C, N, and P contents and their stoichiometric ratios in various organs of *P. yunnanensis*.

Parameter	Age	Organ	Minimum	Maximum	Mean	Standard deviation	Coefficient of variation(%)
C/g·kg^-1^	young	Fine root	188.10	587.40	416.03	16.73	24
		Stem	151.80	587.40	427.72	15.99	22
		Needle	326.70	613.80	458.24	10.77	14
		Branch	184.80	567.60	416.08	16.17	23
	middle	Fine root	237.6	561	402.51	15.99	24
		Stem	122.1	570.9	421.94	15.53	22
		Needle	333.3	567.6	460.53	10.47	14
		Branch	244.2	587.4	446.1	12.36	17
N/g·kg^-1^	young	Fine root	0.45	3.33	2.06	0.11	31
		Stem	0.47	1.08	0.81	0.02	18
		Needle	1.85	6.70	4.13	0.21	31
		Branch	0.81	2.69	1.64	0.10	36
	middle	Fine root	1.05	3.39	2.04	0.09	27
		Stem	0.49	3.18	0.94	0.09	59
		Needle	0.81	4.94	2.63	0.2	45
		Branch	0.59	7.54	1.99	0.25	75
P/g·kg^-1^	young	Fine root	0.19	2.14	1.13	0.08	41
		Stem	0.05	1.34	0.64	0.04	39
		Needle	0.18	2.45	1.06	0.09	50
		Branch	0.11	7.68	1.17	0.22	112
	middle	Fine root	0.37	1.93	1.22	0.07	36
		Stem	0.22	2.95	0.75	0.08	66
		Needle	0.1	3.53	1.35	0.12	55
		Branch	0.01	2.83	0.86	0.09	62
C:N	young	Fine root	104.84	1007.47	232.56	25.69	66
		Stem	167.89	1036.13	553.03	31.50	34
		Needle	68.91	260.51	121.85	7.24	36
		Branch	144.94	522.36	280.40	17.75	38
	middle	Fine root	75.83	504.06	217.19	15.8	44
		Stem	124.71	1133.74	528.44	35.69	41
		Needle	67.53	614.89	248	30.33	73
		Branch	42.46	807.59	323.16	30.59	57
C:P	young	Fine root	148.96	2342.39	493.61	68.13	83
		Stem	224.30	11365.67	1024.07	300.97	176
		Needle	156.26	2902.48	695.82	115.18	99
		Branch	71.33	4550.48	783.97	158.92	122
	middle	Fine root	133.11	1180.01	404.13	39.55	59
		Stem	68.32	1552.82	730.03	56.35	46
		Needle	116.06	4325.36	617.15	142.98	139
		Branch	184.21	31169.03	1860.22	882.87	285
N:P	young	Fine root	0.66	9.27	2.27	0.27	72
		Stem	0.37	15.39	1.76	0.40	135
		Needle	1.14	32.42	6.98	1.42	122
		Branch	0.27	8.71	2.59	0.37	87
	middle	Fine root	0.68	3.51	1.87	0.12	4
		Stem	0.33	4.89	1.52	0.14	55
		Needle	0.33	24.82	3.23	0.82	153
		Branch	0.38	67.78	5.72	2.01	211

Strong variation was detected in the P content and the C:P and N:P ratios (CV, 112%, 285%, and 211%, respectively) of the branches and the C:P and N:P ratios (CV, 139% and 153%, respectively) of the needles in the middle-aged stands. Strong variation was observed in the C:P and N:P ratios of the stems (CV 176% and 135%, respectively), the C:P ratio of the branches (CV 122%), and the N:P ratio of the leaves (CV 122%) in the young stands.

### 3.3 Seasonal variations in the C, N, and P contents in various organs of *P. yunnanensis*

**Figure 1:**
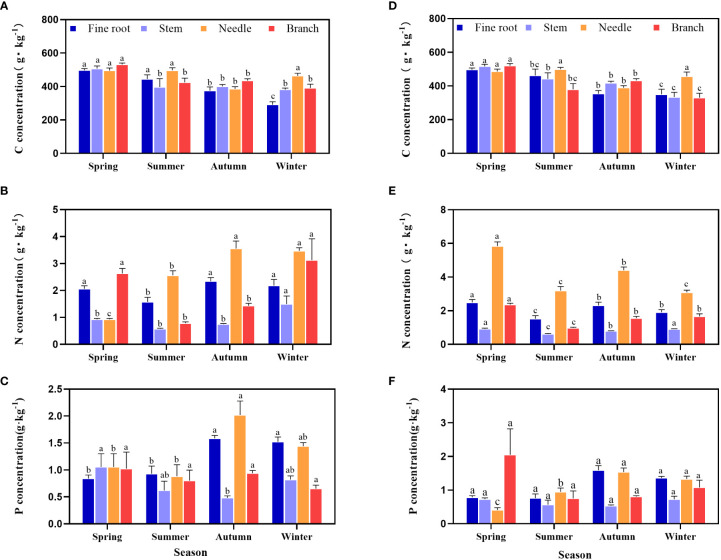
Seasonal dynamics in the C, N, and P contents of various organs in the middle **(A-C)** and young-growth **(D-F)** *P. yunnanensis* stands. Different lowercase letters indicate significant differences between different seasons for the same organ (*P* < 0.05).

The C, N, and P contents of the *P. yunnanensis* organs varied seasonally with different characteristics (**Figure 1**). The C content of root, stem and branch did not change significantly between seasons. The C content of needles was highest during the summer and the winter (**Figures 1A–D**). The N content of needles in the young forests was higher than that of other organs throughout the year (**Figure 1E**). The middle-aged stands had the highest P content in roots during summer and winter, as well as in stems during summer, and in needles during autumn (**Figure 1C**).

### 3.4 Seasonal variation in the C: N: P ratio of various P. yunnanensis organs

**Figure 2:**
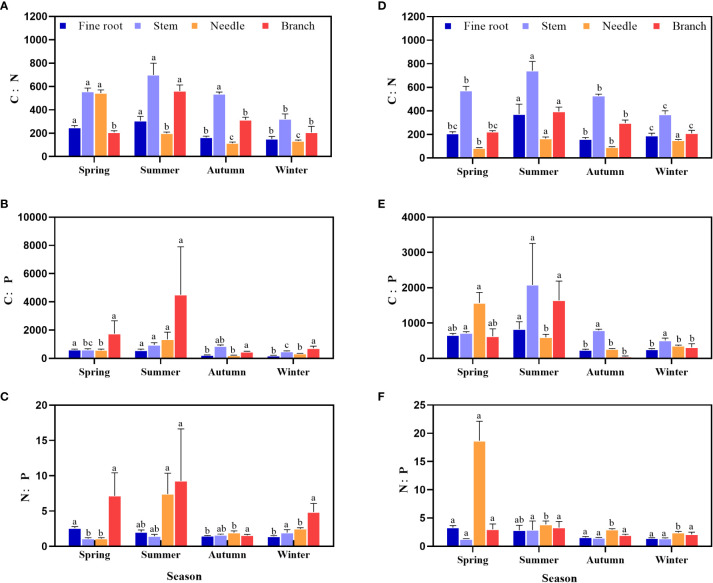
Seasonal dynamics of the C:N:P contents in various organs of middle **(A–C)** and young growth **(D–F)** P. yunnanensis.Different lowercase letters indicate significant differences between different seasons for the same organ (P < 0.05).

The C:N:P stoichiometry ratio in the middle and young stands of *P. yunnanenis* showed clear seasonal changes (**Figure 2**), from spring to winter, the C:N ratio of the stems, branches and fine roots in the middle and young stands increased and then decreased. Throughout the growing season, the C:N ratio of the stems was higher than that of the roots, needles, and branches in the middle and young stands (**Figures 2A–D**). The C:P ratio of needles was highest during the spring and higher than that of roots, needles, in the young forests (**Figure 2E**). The N:P ratio in stems from the middle forests increased, and stems in the young forests showed an increasing and then a decreasing trend. Furthermore, the N:P ratio of needles was higher than that of other organs throughout the growing season in the young forests (**Figure 2**).

### 3.5 Correlation between C, N, and P contents of each organ and their ratios

**Figure 3:**
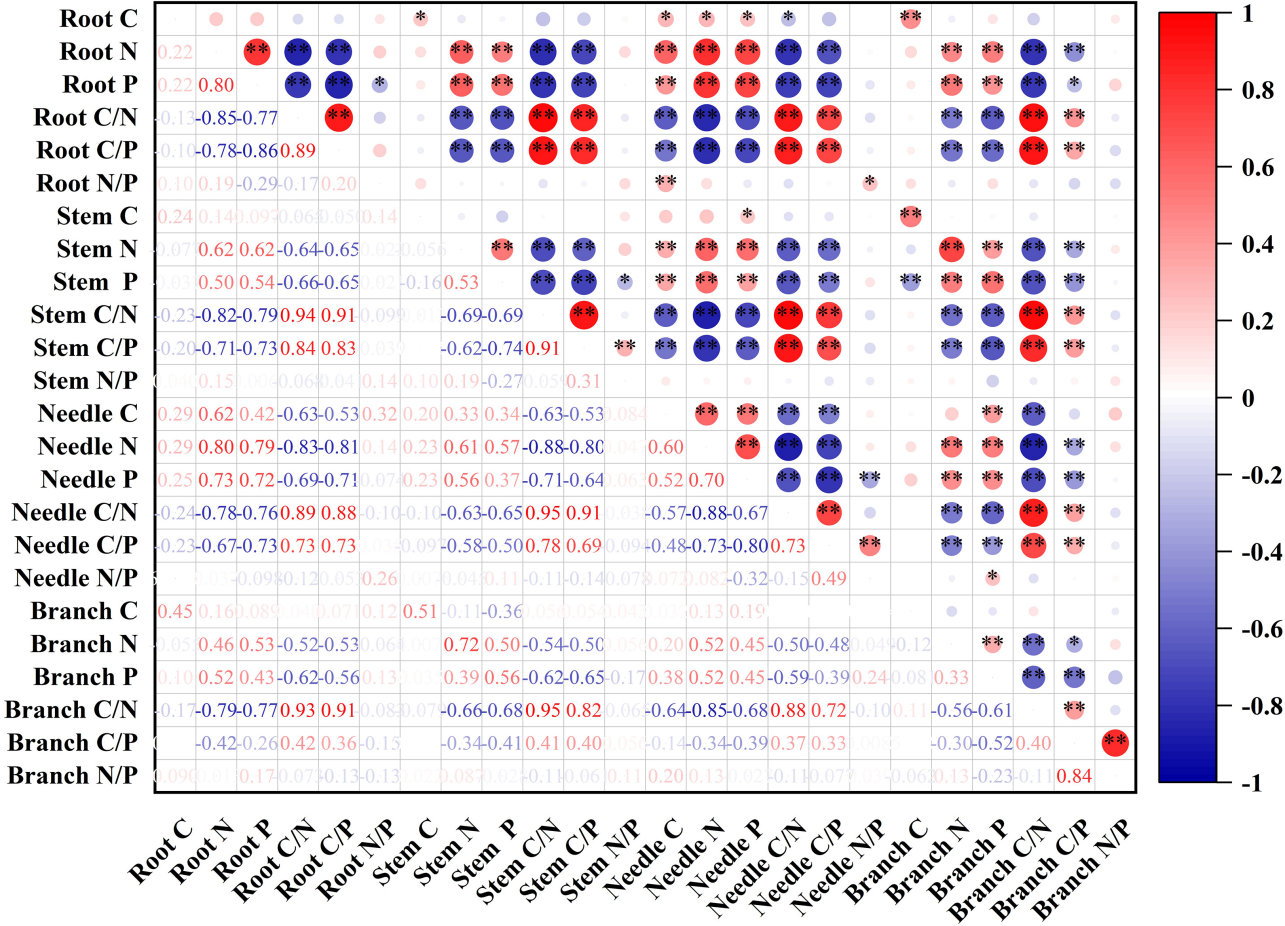
Correlation coefficients between organ C, N, and P contents and their stoichiometric ratios.*P < 0.05, **P < 0.01.

The N and P contents of fine roots were highly significantly and positively correlated with the N and P contents of stems, needles and branches. The C:N and C:P ratios of the fine roots were highly significantly and negatively correlated with the N and P contents of the stems, needles, and branches, whereas the C:N and C:P ratios of the stems and needles, and the C:N ratio of branches were all highly significantly and positively correlated (**Figure 3**).

### 3.6 Allometric growth relationships of C, N, and P in different organs

**Figure 4:**
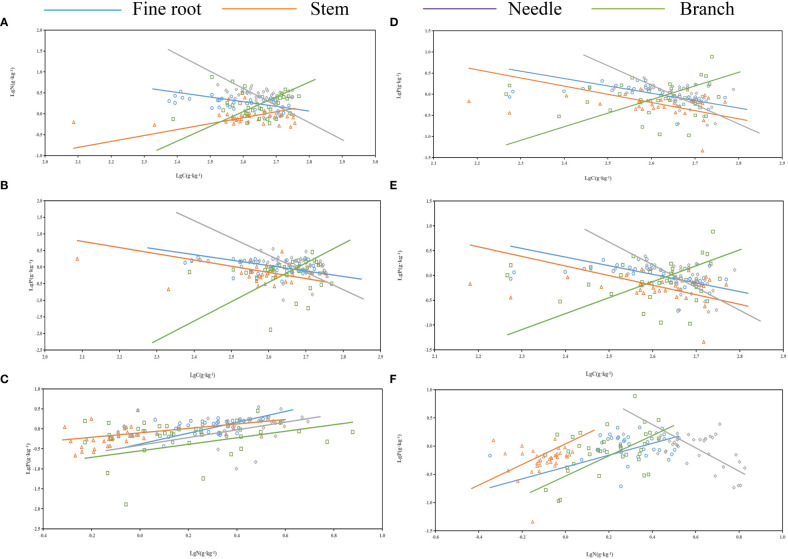
Standardized major analysis (SMA) of carbon (C), nitrogen (N), and phosphorus (P) contents of different organs in *P. yunnanensis*. **(A-C)** and **(D-F)** represented middle-aged and young stands, respectively.

As shown in **Table 3** and **Figure 4**, no significant allometric growth relationship existed for the P-C of branches and stems in the middle and young aged stands, and a significant allometric growth relationship existed for N-P of needles in the young stands. The allometric exponent of the fine roots, needle C-P, and needle N-P were significantly less than 1, but that of stem N-P was significantly greater than 1. The allometric exponent of needle C-N and fine root C-P in the middle-aged forest were significantly less than 1, on the contrary, which of fine root N-P were significantly greater than 1. N-P was positively correlated in all organs of the middle-aged forest (**Figure 4C**), and there was a common slope of 1.418, which was significantly different from 1.0, with a 95% confidence interval of 1.21-1.67.

**Table 3:** The allometric relationships between the C, N, and P contents of different organs in *P. yunnanensis*.

logY vs.logX	Age	Organ	Slope	95%CI	n	Intercept	R^2^	F	P_-1.0_	Type
logN vs.logC	young	Fine root	1.32	[0.94,1.85]	36	-3.15	0.00	2.68	0.11	I
		Stem	-0.70	[-0.99,-0.50]	36	1.74	0.02	4.52	0.04	A
		Needle	2.18	[1.55,3.07]	36	-5.20	0.00	25.18	0.00	A
		Branch	1.36	[1.00,1.87]	36	-3.37	0.16	4.01^*^	0.05	I
logP vs.logC		Fine root	-1.74	[-2.39,-1.27]	36	4.55	0.15	13.68^*^	0.00	A
		Stem	-1.94	[-2.73,-1.38]	36	4.83	0.01	17.29	0.00	A
		Needle	-4.59	[-6.22,-3.39]	36	12.15	0.22	207.74^**^	0.00	A
		Branch	3.22	[2.29,4.54]	36	-8.49	0.00	72.22	0.00	I
logN vs.logP		Fine root	1.32	[0.94,1.86]	36	-0.37	0.02	2.75	0.11	I
		Stem	2.76	[1.97,3.86]	36	0.04	0.03	50.44	0.00	A
		Needle	-2.11	[-2.81,-1.58]	36	1.21	0.31	32.661^**^	0.00	A
		Branch	2.36	[1.74,3.22]	36	-0.53	0.19	39.711^*^	0.00	A
logN vs.logC	middle	Fine root	-1.12	[-1.56,-0.81]	36	3.19	0.07	0.47	0.50	I
		Stem	1.43	[1.01,2.01]	36	-3.79	0.00	4.47	0.04	A
		Needle	-4.09	[-.5.58,-3.00]	36	11.24	0.18	153.67^*^	0.00	A
		Branch	3.53	[2.51,4.97]	36	-9.13	0.00	90.08	0.0	A
logP vs.logC		Fine root	-1.65	[-.2.25,-1.20]	36	4.32	0.16	10.861^*^	0.00	A
		Stem	-1.88	[-2.64,-1.33]	36	4.70	0.00	15.30	0.00	A
		Needle	-5.20	[-7.23,-3.74]	36	13.87	0.08	230.48	0.00	A
		Branch	5.83	[4.15,8.21]	36	-15.62	0.00	272.60	0.00	A
logN vs.logP		Fine root	1.47	[1.08,2.00]	36	-0.38	0.20	6.594^*^	0.02	A
		Stem	1.32	[0.95,1.82	36	-0.10	0.11	2.92	0.10	I
		Needle	1.27	[0.91,1.77]	36	-0.41	0.06	2.12	0.15	I
		Branch	1.65	[1.18,2.31]	36	-0.55	0.03	9.62	0.00	A

P_-1.0_ indicates a significant difference between the estimated model slope and the theoretical value of 1.0, A indicates an allometric growth relationship, and I indicates an isometric growth relationship.

### 3.7 Differences in the allometric relationships among C, N, and P contents of the needles and fine roots

**Figure 5:**
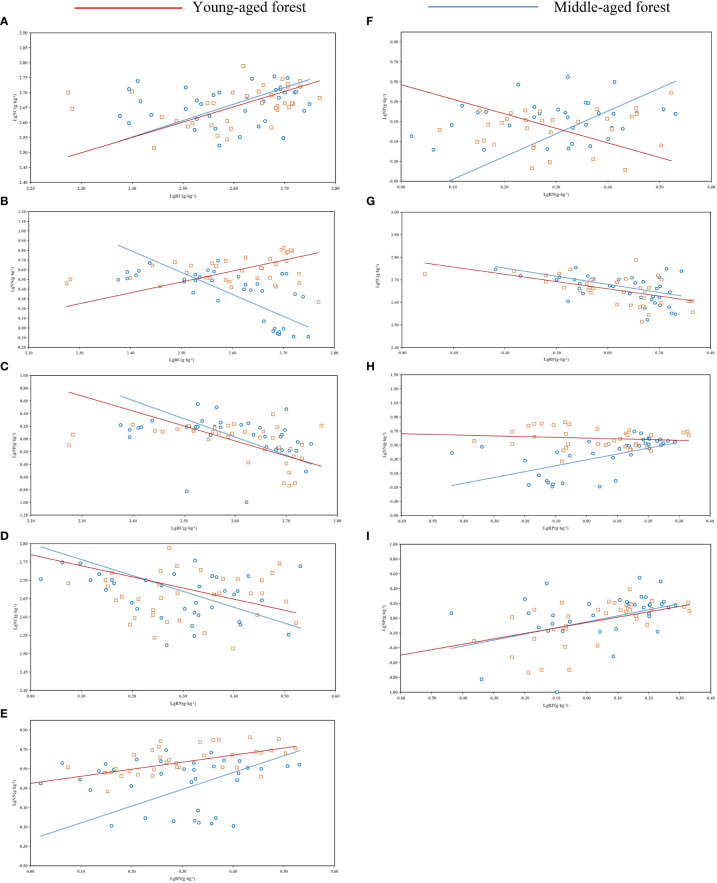
Standardized major analysis (SMA) of carbon (C), nitrogen (N), and phosphorus (P) contents in the needles and fine roots of *P. yunnanensis.* A-I represented the allometric growth relationship between needle and fine root C, N, and P content.

The C, N, and P contents of the needles and fine roots of *P. yunnanensis* varied among stands, reflecting their different equilibrium relationships (**Table 4**). Significant allometric relationships were observed between the C content of the needles and fine root C and P contents, respectively, in addition, highly significant allometric relationships were observed between the N content of the needles and fine root C and P contents, respectively, in young stands. A significant allometric relationship was detected between the P content of the needles and fine root, and a highly significant allometric relationship was observed between the C content of needles and the fine root N content, and the P content of needles and the fine root C and N contents, respectively, in the middle-aged forest. The C content of fine roots and the N content of needles were negatively correlated in the middle-aged forest, and positively correlated in the young forest (**Figure 5F**). The N content of fine roots and the P content of needles were positively correlated in middle-aged forests and negatively correlated in young forests (**Figure 5H**). The allometric relationships of C, N and P contents in needles and fine roots in young and middle-aged forests were the same, which were positively correlated (**Figures 5A, C, E, I**). The allometric relationship between P content in needles and C content in fine roots, and the allometric relationship between C content in needles and N and P content in fine roots were the same, which were all negatively correlated (**Figures 5B, G, D**).

**Table 4:** SMA analysis of C, N, and P contents in the needles and fine roots of *P. yunnanensis*.

Index	Age	Slope	95%CI	Intercept	R^2^
Needle C-Fine root C	young	0.51	[0.37,0.71]	1.32	0.11^*^
	middle	0.55	[0.73,1.72]	1.23	0.03
Needle C-Fine root N	young	1.12	[0.81,1.56]	-2.33	0.06
	middle	-2.26	[4.67,7.78]	6.23	0.42^**^
Needle C-Fine root P	young	-2.36	[-3.23,-1.73]	6.11	0.17^**^
	middle	-2.88	[5.01,9.99]	7.50	0.09
Needle N-Fine root C	young	-0.39	[-0.55,-0.28]	2.77	0.00
	middle	-0.49	[-0.68,-0.36]	2.80	0.11^*^
Needle N-Fine root N	young	0.85	[0.62,1.17]	0.35	0.14^*^
	middle	2.02	[-1.44,2.84]	-0.23	0.00
Needle N-Fine root P	young	-1.79	[-2.51,-1.28]	0.47	0.04
	middle	2.57	[1.84,3.58]	-0.71	0.06
Needle P-Fine root C	young	-0.30	[-0.39,-0.22]	2.66	0.29^**^
	middle	-0.34	[-0.45,-0.25]	2.68	0.23^**^
Needle P-Fine root N	young	-0.64	[-0.90,-0.46]	0.60	0.03
	middle	1.38	[1.04,1.82]	0.29	0.34^**^
Needle P-Fine root P	young	1.36	[1.02,1.81]	-0.06	0.29^**^
	middle	1.75	[1.28,2.39]	-0.05	0.18^**^

*P < 0.05; **P < 0.01.

## 4 Discussion

### 4.1 Analysis of limiting elements for the growth of *P. yunnanensis* stands

*P. yunnanensis* is mainly distributed in southwestern China, where the largest distribution area is in Yunnan Province. The mean C content of *P. yunnanensis* needles in the middle and young stands in this study was 459.39 g·kg^-1^, which was close to the global average C content of plant leaves and slightly higher than the C content of *P. yunnanensis* needles at the Zhanyi Township (Cheng et al., 2022) and in Huize County (Liu et al., 2020) of Yunnan province. The average N content of *P. yunnanensis* needles was 3.38 g·kg^-1^, which was significantly lower than the global and national average N content of plant leaves, and lower than the N content of *P. yunnanensis* needles at the Zhanyi Township and in Huize County. The P contents of *P. yunnanensis* needles at the study sites in Yiliang County, Zhanyi Township, and Huize County were all lower than the global and national P content averages for plant leaves (**Table 5**), which may be due to a phosphorus deficiency in terrestrial ecosystems in China compared with other regions, and the deficiency was more severe in Yunnan province(Shen, 1998). The lower N and P contents of *P. yunnanensis* needles in this study than those in Zhanyi Township and Huize County may be due to the lower average annual temperature (Reich and Oleksyn, 2004). The C:N and C:P ratios of the needles in this study and in Huize County and Zhanyi Township in Yunnan province were higher than the global average for plant leaves (23.80 and 300.90, respectively), whereas the N:P ratio was lower than the global average for all plant leaves (13.8). Han et al. (2005) showed that plant growth is mainly restricted by N when the N:P ratio of leaves is < 14 and is mainly restricted by P when the N:P ratio is > 16. Growth was restricted by both N and P if the N:P ratio was 14-16. This observation indicates that *P. yunnanensis* at this study site, Huize County and Zhanyi Township in Yunnan province was mainly restricted by N.

**Table 5:** The C, N, and P concentrations and their stoichiometric ratios in *P. yunnanensis* needles in this study compared to previous studies in Yunnan Province.

Study area	C	N	P	C:N	C:P	N:P	Data source
Yiliang County	459.39	3.38	1.21	184.93	656.49	5.11	This study
Huize County	437.07	4.63	0.80	94.40	546.34	5.79	Liu et al.,2020
Zhanyi Township	438.00	10.5	0.98	41.71	446.94	10.71	Chen et al.,2021
National		18.60	1.21			14.40	Han et al., 2005
Global	464.00	20.10	1.99	23.80	300.90	13.80	Reich et al., 2004

For the data in the table (this study), the average of the four sampling data from spring, summer, autumn and winter was used.

### 4.2 Seasonal dynamics of C, N, and P and their stoichiometric ratios in various organs of *P. yunnanensis*

The season affects the stoichiometric characteristics of the plant chemical elements in 2 ways, the changes in the functional metabolism during growth and development, and the influence of temperature and precipitation on the chemical elements during the growing season (Wright and Westoby, 2003; Yang and Luo, 2011). In this study, the C, N, and P contents of *P. yunnanensis* and their stoichiometric ratios were mainly affected by season and organ, but were less affected by age (**Table 2**), probably because there was some similarity in the uptake and utilization of elements based on the growth and differentiation of *P. yunnanensis* organs in the different age stands, The result showed a difference with the study of *Metasequoia glyptostroboides*, which showed significant differences in stoichiometric ratios between young and middle-aged stands (Zhang et al., 2018).

The N content in the roots of *P. yunnanensis* was high during the winter, but decreased during the summer (**Figure 1**). This is due to the large amount of nutrient required by growth of new roots emergence and rapid growth of above-ground plants. The N and P contents of the fine roots increased from the summer to autumn period because plant growth is accelerated, the root system needs to absorb large amounts of nutrients to supply growth. This conclusion is consistent with the results of Zhao et al. (2014) on the N and P contents in fine roots of a 20-year-old northern Chinese larch (*Larix principis-rupprechtii*) plantation in the Qinling Mountains during different seasons. In the present study, young and middle-aged forests the C content of fine roots decreased continuously throughout the growing season, the N and P contents in fine roots decreased from autumn to winter (**Figure 1**), probably due to the flow of N and P from the fine roots to the branches (An et al., 2011).

The stem is a conducting organ that connects underground absorption organs to the aboveground assimilation tissues (Zhao et al. 2014). The present study showed that the N and P contents were minimal in stems (**Figure 1**), which is consistent with the results of Zhao et al. (2014). The C content in the stems of the middle-aged forests increased may due to enhanced photosynthesis, while the C content in the young forests decreased during the autumn, probably due to the relatively slow growth of the middle-aged forests, reduced dry matter synthesis, and lower C demand, so the consumption of C by the stems of the middle-aged forests decreased. Plant branches are a link between the carbon-supplying organs (leaves) and the carbon-demanding organs (stems) and are mainly composed of C-rich polysaccharides, such as lignin and cellulose, which coordinate the energy for the plant. This structural property determines their high C concentration (Kong et al., 2020). In this study, the C content of the middle and young aged forest branches was higher than that of other organs during the early and peak growth (**Figures 1A–D**), which is consistent with the results of He et al. (2017) on the C, N, and P contents of different organs of narrow-leaved freshbush (*Sibiraea angustata*) shrubs in the eastern Tibetan Plateau.

Needles are the main organ for mineral uptake aboveground and an important reservoir of nutrients for *P. yunnanensis* during the growing season. The results of this study show that the C content of *P. yunnanensis* needles increased gradually during the spring and highest levels during the summer (**Figure 1**), probably due to the accumulation of C in the leaves as a result of increased photosynthesis and vigorous leaf nutrient metabolism in summer due to the higher ambient temperatures. At the winter, leaf C content rebounded significantly, probably due to the increased C storage capacity and the accumulation rate as a result of limiting plant N and P supply (Herbert et al., 2003). During the spring to summer, N in the needles from the middle and young aged stands and P in the needles from the middle-aged stands may have been influenced by dilution effects (Townsend et al., 2007), as their contents gradually decreased with time, while the N and P contents in needles gradually increased by the autumn. N and P storage in needles increased at the be beginning, while the N content of the young stands reached its lowest level at the end of growth (**Figure 1E**), which was contrary to the result reported by Li et al. (2017) on the lowest N content of needles in northern China larch (*L. principis-rupprechtii Mayr*.) in the Qinling region at the end of growth.

The C:N and C:P ratios represent nutrient use efficiency and the assimilation response of the plant, respectively (Ågren, 2004; Vrede et al., 2004), while the N:P ratio represents the nutrient limitation status (Herbert et al., 2003). In this study, the C:N and C:P ratios of all organs in the middle and young stands decreased significantly from the summer to the autumn (**Figures 2A–E**), indicating that the utilization of N and P decreased during peak growth, which is different from Li et al. (2017) on needle C:N and C:P ratios of northern Chinese larch (*L. principis-rupprechtii Mayr*.) in the Qinling region. C:N and C:P increased with the growing season, probably because of the different stand conditions and tree species. The N:P ratio of young forest needles in this study decreased with season (**Figure 2F**), which is similar to the results of Liu et al. (2015) on alkali ponies (*Suaeda salsa*) in the coastal wetlands of the Yellow River Delta, China. The increase in the C:N ratios in all organs indicates an increase in N utilization efficiency, and the increases in the C:P ratios in stems and branches indicate an increase in P utilization efficiency during this summer (Ågren, 2004; An et al., 2011). The variation in the C:N ratio was small, and the variations in the C, N, and P contents and their stoichiometric ratios in *P. yunnanensis* were mainly affected by organ and season (**Table 2**), suggesting the specificity of the uptake and utilization of elements in the organ level.

### 4.3 Distribution patterns of C, N, P and their stoichiometric ratios in different organs of P. yunnanensis

Differences in nutrient requirements of different organs in the same tree species can lead to variability in the distribution of elements among different organs, and such differences reflect how plants adapt to specific environments (Aerts and Chapin, 1999). In the present study, the C content of all organs of *P. yunnanensis* in the middle and young stands was greater in stems than needles at peak growth (**Figures 1A–D**), which was similar to the results of Liu et al. (2015) on alkali ponies (*S. salsa)* in the coastal wetlands of the Yellow River Delta, China. N and P contents were in the order needles > fine roots > branches > stems at peak growth (**Figures 1B–F**), which was consistent with the results of Zhao et al. (2014) on the C, N, and P contents of northern Chinese larch (*L. principis-rupprechtii*) roots and leaves. The N and P contents of needles were higher than those of fine roots and stems, and the C contents of branches and stems were higher than those of needles and fine roots because 1) leaves are the main photosynthetic organs utilizing nutrients, and their nutrient contents reflect the nutritional status of the tree (Zhang et al., 2008). 2) Branches and stems transport nutrients, and play a supporting role, and C is an essential element for macromolecules, such as the cytoskeleton (He et al., 2017; Feng et al., 2019). At peak growth, the C:N ratio of each organ in the *P. yunnanensis* middle and young-growth forests was in the order stems > branches > roots > needles, and the C:P ratio was highest in the stems, while the N:P ratio was highest in the needles (**Figure 2**), probably because C content is more stable in organs relative to other elements, so the levels of N and P determined the magnitudes of the C:N and C:P ratios (Feng et al., 2019).

### 4.4 Allometric exponent relationships among C, N, and P in the different organs of P. yunnanensis

The N-C, P-C, and P-N allometric relationships describe a random constraint strategy for C:N:P (Li et al., 2017). In this study, P-C of branches and stems of the middle and young stands did not have significant allometric growth relationships, which was similar to the results of Liu et al. (2015) on the allometric growth of C-P in different organs of alkali ponies (*Suaeda salsa*), indicating that the respective nutrient allocation to branches and stems was similar, probably due to the different consistency of the nutrient utilization pattern of *P. yunnanensis* branches and stems. The N-P of young forest needles had a significant allometric growth relationship, which was consistent with the findings of Wang et al. (2015) on desert halophytes in arid saline environments of the northwest, indicating that their growth was significantly affected by the nutrient concentrations. The present study showed that there was a common slope in the N-P allometric growth in all organs of the middle-aged forests, indicating a strong correlation between N and P contents (Reich et al., 2010). This finding reflects that the N and P biochemical pathways related to basic cell structure and function differed between different stands age.

Some allometric relationships exist between many of the traits of above and below-ground plant organs, such as specific leaf area to specific root length (Li and Bao, 2015) and the leaf mass ratio to the fine root mass ratio (Imada et al., 2010). These allometric relationships provide an important basis for coordinating the growth and development of different organs (Meng et al., 2018). This study found significant allometric relationships among C, N, and P contents in needles and roots from the middle and young stands of *P. yunnanensis*, but there was no common slope, indicating a difference in their rate of change. The C and N contents of the needles and leaves in the young stands had significant allometric relationships with the C, N, and P contents of the fine roots, while the C, N, and P contents of the fine roots had significant allometric relationships with the C and N contents of the needles in the middle-aged stands, indicating that the coordination of nutrients between the needles and leaves and fine roots of *P. yunnanensis* in different age stands differed, mainly in the utilization of P, which was allocated to roots in the young stands and more to needles in the middle-aged stands.

## 5 Conclusions

C, N, and P contents and their stoichiometric ratios did not differ significantly in the middle and young stands of *P. yunnanensis*; however, they were significantly affected by the season and organ as well as by the interaction between them. The C contents of stems and branches of *P. yunnanensis* increased then decreased from spring to winter. The N content of stems and branches decreased first and then increased, whereas the P content of the fine roots increased first and then decreased, indicating that *P. yunnanensis* has significantly different nutrient allocations in different organs.

According to the content of N, P and ratio in needles, the growth of *P. yunnanensis* in the study area is limited by N element. It is recommended that N fertilizer be added appropriately to manage *P. yunnanensis* stands and promote healthy and rapid growth.

There is variability in the distribution of nutrient elements among organs of *P. yunnanensis* in different age stands. No significant allometric growth relationship existed for the P-C of branches and stems in the middle and young aged stands, indicating a similarity in nutrient allocation between branches and stems in different age stands. A significant allometric growth relationship existed for N-P of needles in the young stands, indicating significant differences in their rates of change in different age stands.

In studying the relationship between the allometric growth of C, N and P in needles and fine roots, we found that P were allocated more to needles in middle-aged stands and fine roots in young stands, indicating that the nutrient coordination between the needles and fine roots of *P. yunnanensis* differed among stands of the different ages.

In this study, the C, N, and P stoichiometry of the different *P. yunnanensis* organs were discussed in depth, but only two stand ages were selected for this study, which was a limitation, so more stand ages should be selected for future observations.

## Data availability statement

The raw data supporting the conclusions of this article will be made available by the authors, without undue reservation.

## Author contributions

SL wrote the manuscript, JW designed the experiments, provided critical revisions and final approval of the article. DW, CL, HJ and TT carried out the experiments and run the data. All authors contributed to the article and approved the submitted version.
